# Age-related changes in the radiologic findings of lobular endocervical glandular hyperplasia: a multicenter study

**DOI:** 10.1007/s11604-025-01748-y

**Published:** 2025-02-14

**Authors:** Ayumi Ohya, Takuya Fukuzawa, Yuki Himoto, Aki Kido, Takahiro Tsuboyama, Nao Kikkawa, Hideyuki Fukui, Yuko Iraha, Kimiteru Ito, Yasunari Fujinaga

**Affiliations:** 1 Department of Radiology, Shinshu University School of Medicine, 3-1-1 Asahi, Matsumoto, 390-8621 Japan; 2 Department of Diagnostic Imaging and Nuclear Medicine, Kyoto University Graduate School of Medicine, Kyoto, Japan; 3 Department of Radiology, Osaka University Graduate School of Medicine, Osaka, Japan; 4 Departmant of Diagnostic Radiology, National Cancer Center Hospital, Tokyo, Japan; 5 Department of Radiology, Graduate School of Medical Science, University of the Ryukyus, Ginowan, Japan

**Keywords:** Cervix uteri, Lobular endocervical glandular hyperplasia, Magnetic resonance imaging, Cosmos pattern

## Abstract

**Purpose:**

To investigate the age-related changes in magnetic resonance imaging (MRI) findings of lobular endocervical glandular hyperplasia (LEGH) during long-term follow-up.

**Materials and methods:**

This multicenter study included 91 patients who underwent preoperative MRI and had a histopathological diagnosis of LEGH, atypical LEGH, or adenocarcinoma in situ (AIS) with LEGH after surgical resection. Thirty patients underwent follow-up MRIs at intervals of more than 3 months. According to the age and menopausal status, patients were categorized into four groups: group A, 31–40 years; group B, 41–50 years (premenopausal); group C, more than 50 years (premenopausal); group D, postmenopausal. Differences in the MRI findings (size and morphological pattern) were compared among the four groups.

**Results:**

The lesion volume was the largest in group C and smallest in group D, showing a statistically significant difference (*p* < 0.05). The typical cosmos pattern was seen in 60.0% of group A, 62.2% of group B, 75.0% of group C, and 29.2% of group D. The cosmos pattern was significantly less frequent in postmenopausal patients compared to premenopausal patients (*p* < 0.05). During follow-up, five of 12 individuals in group A exhibited the typical cosmos pattern. Among the seven individuals who did not initially show the cosmos pattern, two later developed the typical cosmos pattern. No changes in the lesion pattern were observed in participants in their 40 s up to the premenopausal 50 s. From the premenopausal 50 s to the postmenopausal period, the cosmos pattern changed to a microcystic pattern in one case of atypical LEGH.

**Conclusions:**

LEGH increases in volume with age until menopause, along with an increasing frequency of the typical cosmos pattern in MRI. However, after menopause, both the volume of the lesion and frequency of the typical cosmos pattern decrease.

## Introduction

Among the various lesion-forming cysts that can occur in the uterine cervix, lobular endocervical glandular hyperplasia (LEGH) requires special clinical consideration. Although LEGH behaves benignly, LEGH with atypia (atypical LEGH) is a precancerous lesion of HPV-independent gastric-type adenocarcinoma (GAS) of the cervix, which has a poor prognosis [1, 2]. In addition, LEGH itself is not easy to differentiate from some gastric-type adenocarcinomas of the cervix because they appear very similar on magnetic resonance imaging (MRI) [3]. Therefore, once LEGH is diagnosed, unlike other benign cervical cystic lesions such as Nabothian cysts, long-term follow-up is indicated.

In the clinical setting, mucous diagnosis, cytology, and MRI are the important diagnostic modalities for LEGH. LEGH secretes gastric-type mucin because of pyloric gland metaplasia [4, 5]. A kit exists to determine the presence or absence of gastric-type mucin in the cervical mucus [6], although it is not available worldwide. Accurate diagnosis based on cytology may be challenging due to its predilection for sites near the internal os of the cervix [1, 7]. Thus, among the three modalities, MRI is crucial for diagnosis. In a previous study, 87% of LEGH showed an MRI finding called the cosmos pattern [8], which is considered a characteristic MRI finding of LEGH. The cosmos pattern refers to a finding where the central part of the lesion contains small cysts and a small amount of solid components, while relatively larger cysts are present at the periphery. However, in a subsequent report, the frequency of the cosmos pattern in gastric-type mucin-positive cervical lesions was 63.1%, which was lower than that in the initial study [7]. One study has proposed the raspberry-type as a novel MRI finding in atypical LEGH [9]. The raspberry-type refers to an MRI finding where the lesion is composed of a cluster of very small cysts and appears hyperechoic on ultrasound. In the above study, most of the cases presenting with the raspberry-type were postmenopausal [9]. In addition, it has been reported that half of the LEGH cases shrink after menopause [10]. Therefore, just as the uterus changes with aging, LEGH might also show age-related changes in the size or morphology on MRI. Hence, the question arises whether the MRI findings of LEGH differ depending on the age of the patient.

Furthermore, the optimal timing of LEGH treatment has not been established yet. However, considering that GAS has a poor prognosis, surgery seems to be the ideal option for atypical LEGH or LEGH with gastric-type adenocarcinoma in situ (AIS), as these are precancerous lesions, distinct from LEGH. Currently, these precancerous lesions are diagnosed by cytology and cone biopsy. However, since there is always uncertainty about whether the required parts of the lesion near the internal os of the cervix are being sampled, these methods cannot be considered ideal for diagnosing precancerous conditions. Therefore, it would be ideal to determine the presence or absence of atypia using MRI, which can capture the entire LEGH lesion. However, MRI findings of atypical LEGH have not yet been identified. Nevertheless, assuming that the MRI findings of LEGH change with age, it is worth exploring whether MRI can detect differences in how LEGH and atypical LEGH change during follow-up.

To the best of our knowledge, no studies have investigated the differences in the MRI findings of LEGH according to age. Due to the relative rarity of LEGH, the number of cases at a single institution was too small for our study. Therefore, we accumulated surgical cases from five institutions and retrospectively investigated the MRI findings of LEGH in different age groups. In addition, in cases that underwent follow-up, we investigated changes in the MRI findings of LEGH over time, along with the differences in the MRI findings between the LEGH group and atypical LEGH group.

## Materials and methods

### Patient population

**Fig. 1:**
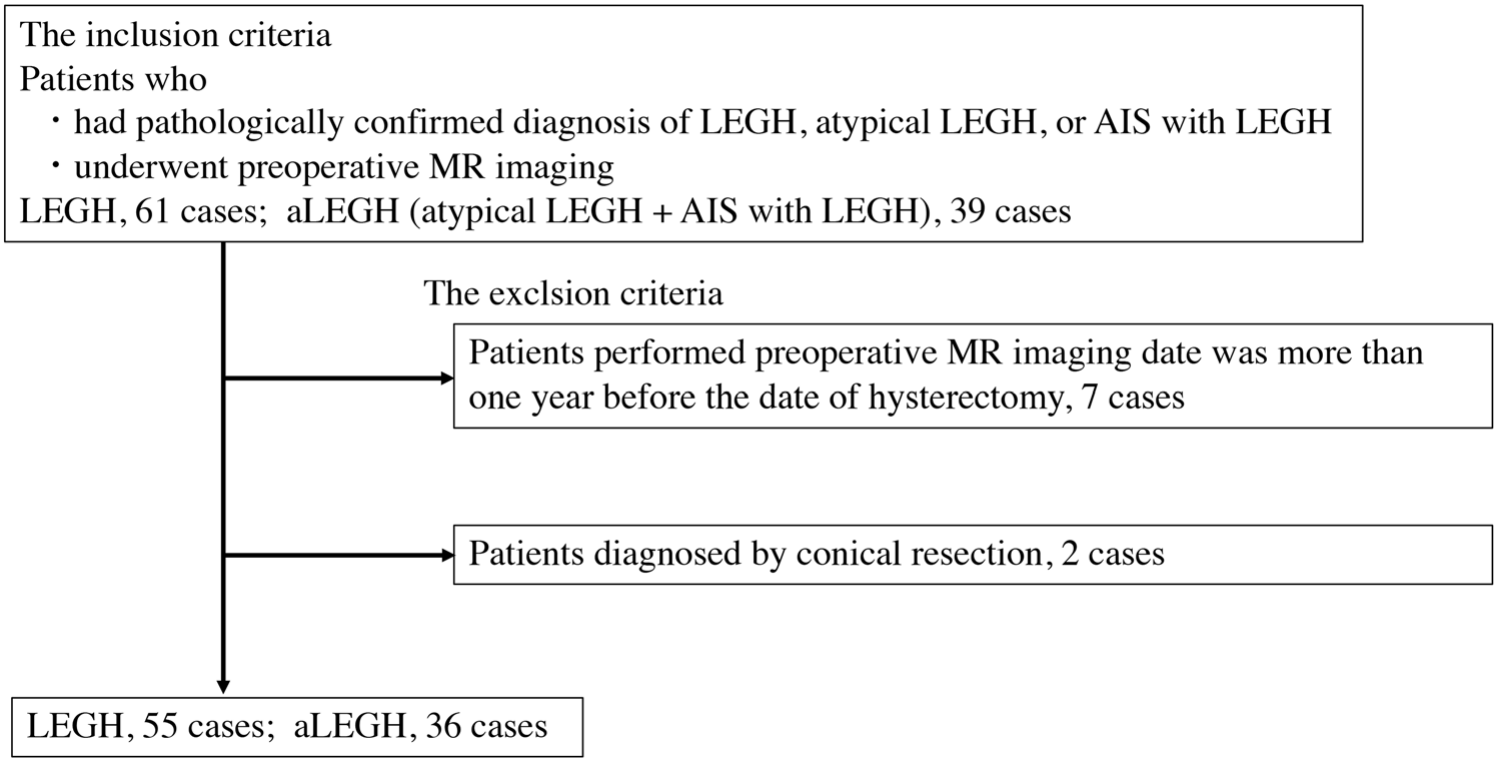
Flowchart of patient enrollment in our study. LEGH, lobular endocervical glandular hyperplasia; AIS, adenocarcinoma in situ; MR, magnetic resonance; aLEGH, atypical LEGH and LEGH with adenocarcinoma in situ

This study was approved by the ethical committee of our institution (no. 2023–048). The requirement for informed consent was waived owing to the retrospective nature of the study. Between January 2000 and December 2022, patients diagnosed with LEGH, atypical LEGH, and AIS with LEGH were identified via the electronic medical records at our hospital and four other institutions: Kyoto university Hospital, National Cancer Center Hospital, Osaka university Hospital, and Ryukyus university Hospital. The flowchart of patient enrollment in this study is summarized in Fig. 1. The inclusion criteria were as follows: patients who had a pathologically confirmed diagnosis of LEGH, atypical LEGH, or AIS with LEGH and underwent preoperative MRI (MRI immediately prior to hysterectomy or conical resection). Patients who underwent preoperative MRI more than 1 year before the date of hysterectomy were excluded. In addition, patients diagnosed by conical resection alone were also excluded because diagnosis by this method is not considered a definitive final diagnosis. Finally, 91 patients were enrolled. Of these, 55 patients (median 49 years; range, 32–75 years) were diagnosed with LEGH (classified into the LEGH group) and 36 patients (median 48.5 years; range, 33–67 years) were diagnosed with atypical LEGH or AIS with LEGH (classified into the aLEGH group). We investigated the patients’ obstetric history, symptoms, preoperative cytology results, and menopausal status. Patient anonymity was ensured in this study.

### MRI analysis

The analyzed MR images were limited to a single set of images taken immediately before hysterectomy or conization for each patient. The median duration between preoperative MR imaging and surgical resection was 61 days (range, 0–220 days) in the LEGH group and 73 days (range, 4–352 days) in the aLEGH group. MRI findings on axial, sagittal, and coronal T2-weighted images (T2WIs) with or without fat suppression were analyzed. The scan parameters and capture devices of MRI varied because this was a retrospective, long-term, and multicenter study. All patients underwent preoperative MRI using 1.5/3.0 Tesla scanners (Philips Electronics N.V., Amsterdam, Holland; GE HealthCare, Chicago, Illinois, USA; Canon Medical Systems Corp., Tochigi, Japan; Siemens Healthcare Diagnostics, Erlangen, Germany); 40 and 51 patients were scanned using 1.5 Tesla and 3.0 Tesla scanners, respectively.

**Fig. 2:**
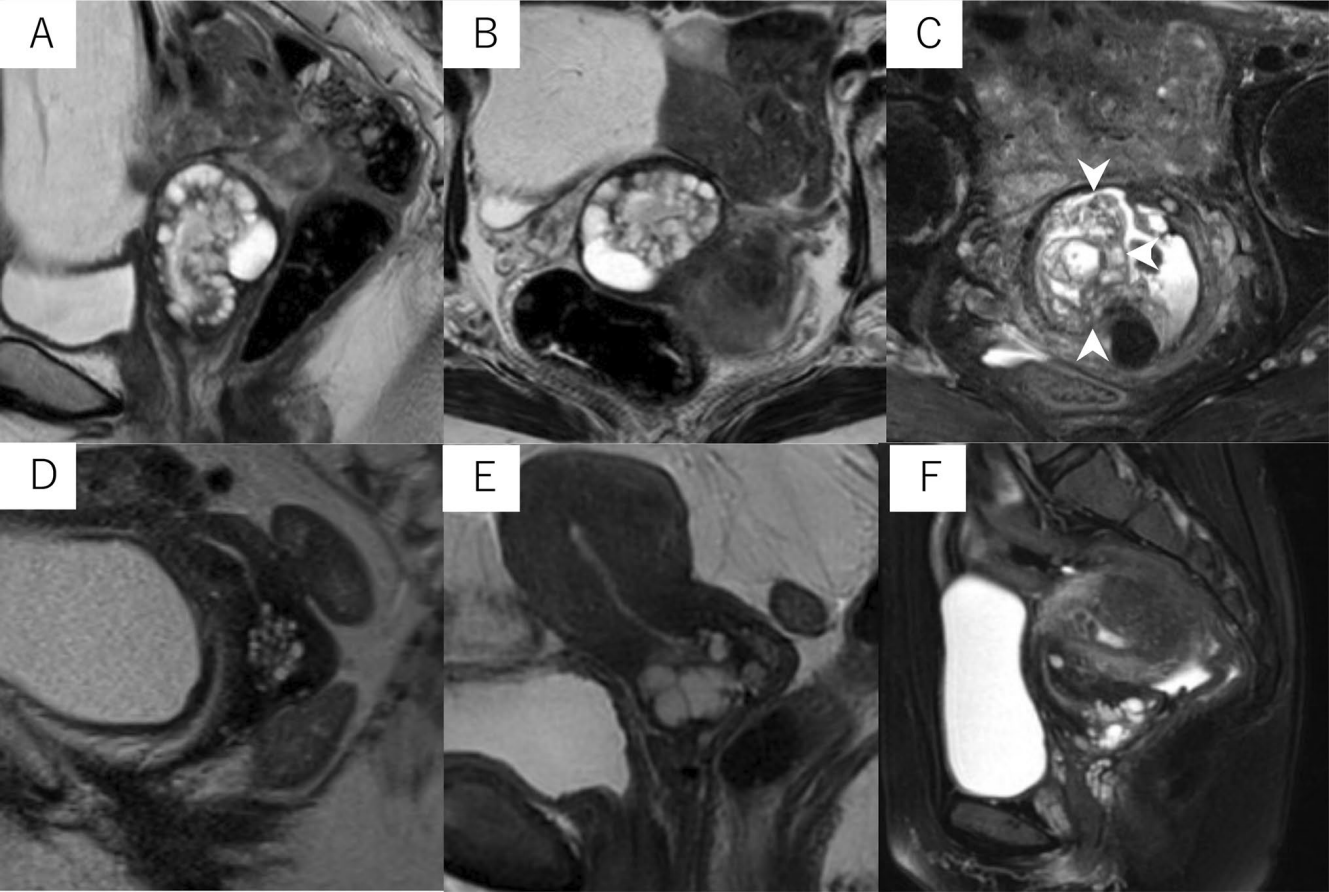
Five types of magnetic resonance imaging features. **A**,**B** Typical cosmos pattern. Sagittal and axial T2-weighted images (T2WIs) shows that the cystic aggregate lesion is mainly located in the upper part of the cervix. Small cysts (< 5 mm) and solid components are visible in the central part of the lesion, surrounded by relatively large cysts (≥ 5 mm). The solid components in the central part show hypointensity similar to that of the cervical stroma or slight hyperintensity compared to the cervical stroma, and are located between the cysts. **C** Atypical cosmos pattern. This lesion meets the criteria for the typical cosmos pattern, with small cysts arranged on the endocervical side and relatively larger cysts on the cervical stromal side. However, it is a polypoid lesion protruding from the right side of the cervical stroma into the endocervical canal (arrowhead). **D** Microcystic pattern. Sagittal T2WI shows that the lesion is located in the upper part of the cervix and composed of many tiny aggregated cysts. **E** Macrocystic pattern. This lesion is mainly made of relatively large cysts on sagittal T2WI. **F** Other pattern. This lesion is composed of variably-sized cysts near the external cervical os

Two experienced radiologists with 22 and 31 years of experience (both blinded to the patients’ clinical information) independently assessed the MRI features of the lesions, specifically the cystic and solid component patterns within the lesions. Disagreements were resolved by discussion. In accordance with a previous study [7], cystic and solid component patterns within the lesions on MRI were classified into five types. The “typical cosmos pattern” (Fig. 2A, B) was characterized by some small cysts (<5 mm) and solid components in the central part of the lesion, surrounded by relatively larger cysts (≥5 mm). Cases in which one of the two radiologists identified a typical cosmos pattern, while the other identified a different pattern and cases presenting with a cosmos pattern but protruding into the cervical lumen in a polypoidal fashion were subclassified into the “atypical cosmos pattern” (Fig. 2C). This definition was adapted during discussions when the two readers disagreed. The “microcystic pattern” (Fig. 2D) was composed of cysts smaller than 5 mm, which were aggregated and scattered throughout the lesion. The “macrocystic pattern” (Fig. 2E) was characterized by cysts larger than 5 mm that exhibited a solitary, scattered, or aggregated pattern. The “unclassifiable pattern” (Fig. 2F) was characterized by cysts clustered predominantly at the external os or those that do not fit any of the above patterns. The lesions were assessed in multiple directions on MRI to minimize the partial volume effect. In addition to the lesion pattern, the lesion size was measured in T2WIs. The volume of the lesion was calculated as the product of the length, width, and height of the lesion, in accordance with the study by Omori et al. [9].

### Follow-up MRI

Thirty of the 91 patients (LEGH; 22 cases, aLEGH; eight cases) underwent two or more MRIs more than 3 months apart. The median number of MRIs was 2.5 (range, 2–10). The minimum interval between the MRI examinations was 3 months and the maximum was approximately 13 years. The median follow-up period was 2050 days, with a minimum of 3 months and maximum of approximately 13 years. The lesion pattern in each follow-up MRI was analyzed.

### Statistical analysis

R software (version 4.3.1) was used for statistical analysis [11]. Differences in the clinical parameters between the LEGH and aLEGH groups were analyzed using the Fisher’s exact test. Patients were categorized into four age groups according to the age and menopausal status as follows: group A, 31–40 years; group B, 41–50 years (premenopausal); group C, older than 50 years (premenopausal); group D, postmenopausal. Lesion volumes were compared among these four groups using the Kruskal–Wallis test, followed by Bonferroni correction for multiple comparisons among all four groups. For comparing the lesion pattern ratios between the premenopausal and postmenopausal groups, the Fisher’s exact test was used. Finally, the agreement between the opinions of the two radiologists was determined by calculating the kappa coefficient. A kappa value of ≤ 0.20 indicated poor agreement; 0.21–0.40, fair agreement; 0.41–0.60, moderate agreement; 0.61–0.80, good agreement; and 0.81–1.00, excellent agreement. A *p* value < 0.05 was considered to indicate statistical significance.

## Results

### Differences in clinical parameters between the LEGH and aLEGH groups

The clinical information of the patients in the LEGH and aLEGH groups is summarized in Table 1. On cytologic examination, 62.4% and 48.5% of the patients in the LEGH and aLEGH groups were found to be negative for intraepithelial lesion or malignancy or Class 1 or 2, respectively. The cytology class tended to be slightly higher in the LEGH group, although the difference was not statistically significant. No significant differences were found in the frequency of symptoms, such as abnormal vaginal discharge, irregular bleeding, and asymptomatic status, between the two groups. Regarding the obstetric history, 40.7% of patients in the LEGH group and 18.2% in the aLEGH group were nulliparous, with statistically significant difference between the two groups (*p* = 0.035).

**Table 1:** Clinical information of the patients

	LEGH group	aLEGH group	*p* value
Symptoms	*n* = 55 (%)	*n* = 36 (%)	
Abnormal vaginal discharge	19 (34.5%)	15 (41.7%)	0.514
Irregular bleeding	10 (18.2%)	8 (22.2%)	0.789
Menstrual disorder	5 (9.1%)	2 (5.6%)	0.699
Asymptomatic	21 (38.2%)	11 (30.6%)	0.507
Others	1 (1.8%)	1 (2.8%)	1
Cytology	*n* = 53	*n* = 33	
NILM	33 (62.3%)	16 (48.5%)	0.264
Abnormal	20 (37.7%)	17 (51.5%)	
History of pregnancy	*n* = 54	*n* = 33	
Nulliparous	22 (40.7%)	6 (18.2%)	0.035

*LEGH* lobular endocervical glandular hyperplasia, *aLEGH* atypical LEGH and LEGH with adenocarcinoma in situ, *NILM* negative for intraepithelial lesion or malignancy

### Differences in the MRI findings by age

**Fig. 3:**
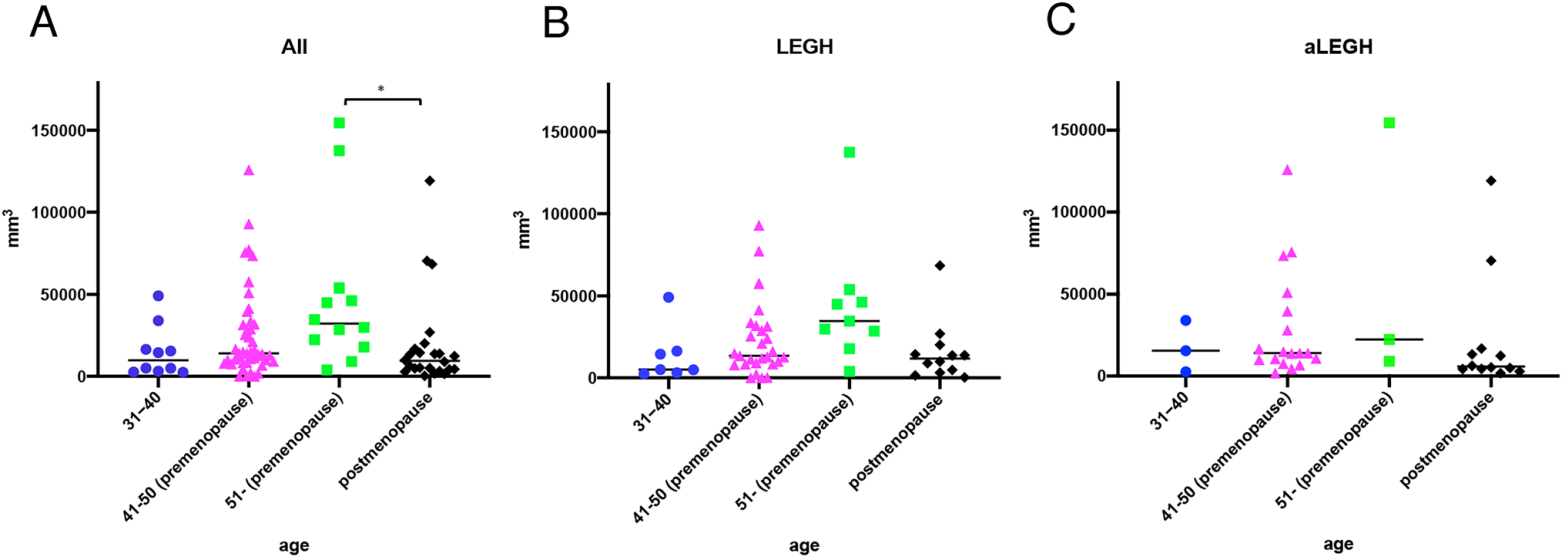
**A** Volume of the lesions by age group. Although there is some overlap in the lesion volume within each age group, the median lesion volume tends to increase from younger age groups to pre-menopause, and decreased significantly after menopause compared to just before menopause. The Kruskal–Wallis and Steel-Dwaas tests were used to compare the lesion volume by age. Overall, a statistically significant difference was found (*p* = 0.014), as well as a significant difference between just before and after menopause (*p* = 0.021). **B**,**C** The shape of the graphs for the LEGH and aLEGH groups is identical, indicating the same trend. LEGH; lobular endocervical glandular hyperplasia, aLEGH; atypical LEGH and adenocarcinoma in situ with LEGH

The relationship between the lesion volume and age in all patients (LEGH and aLEGH) is shown in Fig. 3. The median lesion volume in groups A, B, C, and D was 5100 mm^3^, 14,040 mm^3^, 32,249 mm^3^, and 9433.5 mm^3^, respectively. When multiple comparisons were made among all age groups, a statistically significant difference was observed between group C and D (*p* = 0.019). When the lesion volumes in the LEGH and aLEGH groups were investigated separately, the trend was the same as for the overall cohort, and the median lesion volume was the largest in group C and the smallest in group D. However, when comparing across all age groups, no significant differences were found in the median lesion volume in the LEGH and aLEGH groups.

The percentage of patients with the cosmos pattern in the entire cohort is summarized in Table 2. In all age groups, the cosmos pattern accounted for the largest proportion of MRI findings. Postmenopausal women had the lowest percentage of LEGH lesions showing the typical cosmos pattern (29.2%), which had the same percentage as the microcystic pattern in this age group. In contrast, the percentage of LEGH lesions showing the typical cosmos pattern reached 75% in premenopausal women. In addition, group B had the second highest percentage at 62.2%. On comparing premenopausal and postmenopausal patients, the typical cosmos pattern was significantly more common in premenopausal patients (*p* = 0.0041). When the incidence of the cosmos pattern was examined separately in the LEGH and aLEGH groups, the incidence in both groups tended to be similar to that in the overall cohort (Table 2). On comparing premenopausal and postmenopausal patients in the LEGH group, the proportion of patients with the typical cosmos pattern was significantly higher in premenopausal patients (*p* = 0.045). However, there was no statistically significant difference in the proportion of patients with the typical cosmos pattern between premenopausal and postmenopausal patients in the aLEGH group (*p* = 0.086). The agreement between the two radiologists was good for both the LEGH and aLEGH groups (*κ* = 0.884 and *κ* = 0.712, respectively), but better for the LEGH group.

**Table 2:** MRI features of the lesions

Group (years)	A (31–40)	B (41–50, premenopausal)	C (≧51, premenopausal)	D (postmenopausal)
All (*n* = 91)				
MR features	*n* = 10	*n* = 45	*n* = 12	*n* = 24
Cosmos	6 (60%)	28 (62.2%)	9 (75%)	7 (29.2%)
Atypical cosmos	0 (0%)	0 (0%)	1 (8%)	5 (20.8%)
Microcystic	2 (20%)	8 (17.8%)	0 (0%)	7 (29.2%)
Macrocystic	1 (10%)	5 (11.1%)	1 (8%)	5 (20.8%)
Others	1 (10%)	4 (8.9%)	1 (8%)	0 (0%)
LEGH (*n* = 53)				
MRI features	*n* = 7 (%)	*n* = 25	*n* = 9	*n* = 12
Cosmos	4 (57.1%)	17 (68%)	7 (77.8%)	4 (33.3%)
Atypical cosmos	0 (0%)	0 (0%)	0 (0%)	2 (16.7%)
Microcystic	2 (28.6%)	5 (20%)	0 (0%)	3 (25%)
Macrocystic	1 (14.3%)	2 (8%)	1 (11.1%)	3 (25%)
Others	0 (0%)	1 (4%)	1 (11.1%)	0 (0%)
aLEGH (*n* = 38)				
MRI features	*n* = 3	*n* = 20	*n* = 3	*n* = 12
Cosmos	2 (66.7%)	11 (55%)	2 (66.7%)	3 (25%)
Atypical cosmos	0 (0%)	0 (0%)	1 (33.3%)	3 (25%)
Microcystic	0 (0%)	3 (15%)	0 (0%)	4 (33.3%)
Macrocystic	0 (0%)	3 (15%)	0 (0%)	2 (16.7%)
Others	1 (33.3%)	3 (15%)	0 (0%)	0 (0%)

*MRI* magnetic resonance imaging, *LEGH* lobular endocervical glandular hyperplasia, *aLEGH* atypical LEGH and LEGH with adenocarcinoma in situ

### Changes in LEGH during follow-up

**Fig. 4:**
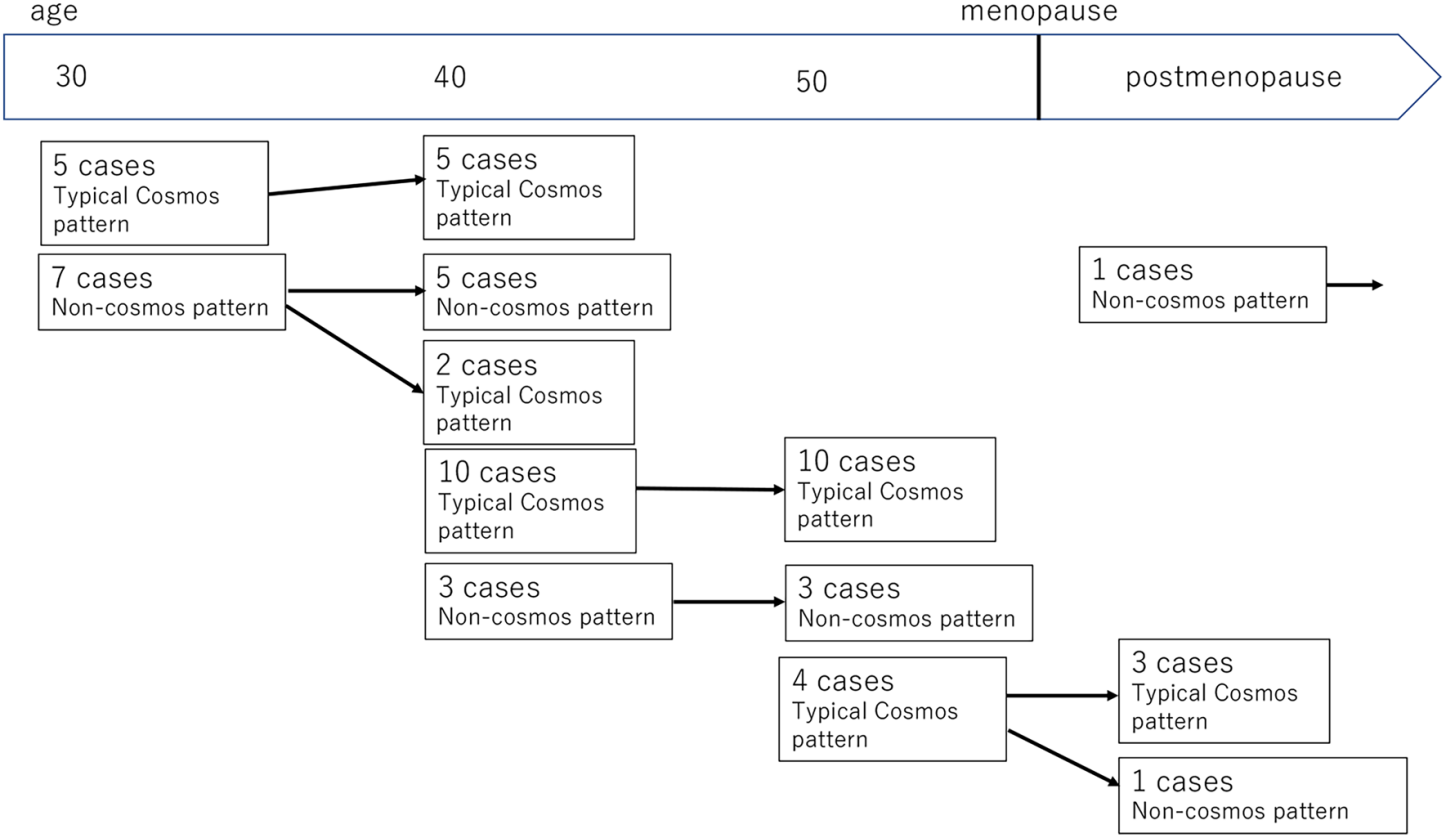
Summary of the changes in the morphologic MRI findings of LEGH during follow-up. In patients in their 30 s and 40 s, five out of 12 cases showed the typical cosmos pattern in the initial MR image with no change over time. Among the remaining seven cases, five showed the non-cosmos pattern in the initial MR image, which remained unchanged until just before surgery, while in two cases, the pattern changed from non-cosmos to cosmos by the time of surgery. From the age of 41 years to pre-menopause, the typical cosmos pattern was observed more frequently (10 out of 13 cases), but no changes in the MRI findings were observed in any case. In the four cases observed from pre-to postmenopause, one case showed a change from the cosmos pattern to non-cosmos pattern (microcystic pattern). For the one case observed postmenopause, no changes were seen in the MRI findings

**Fig. 5:**
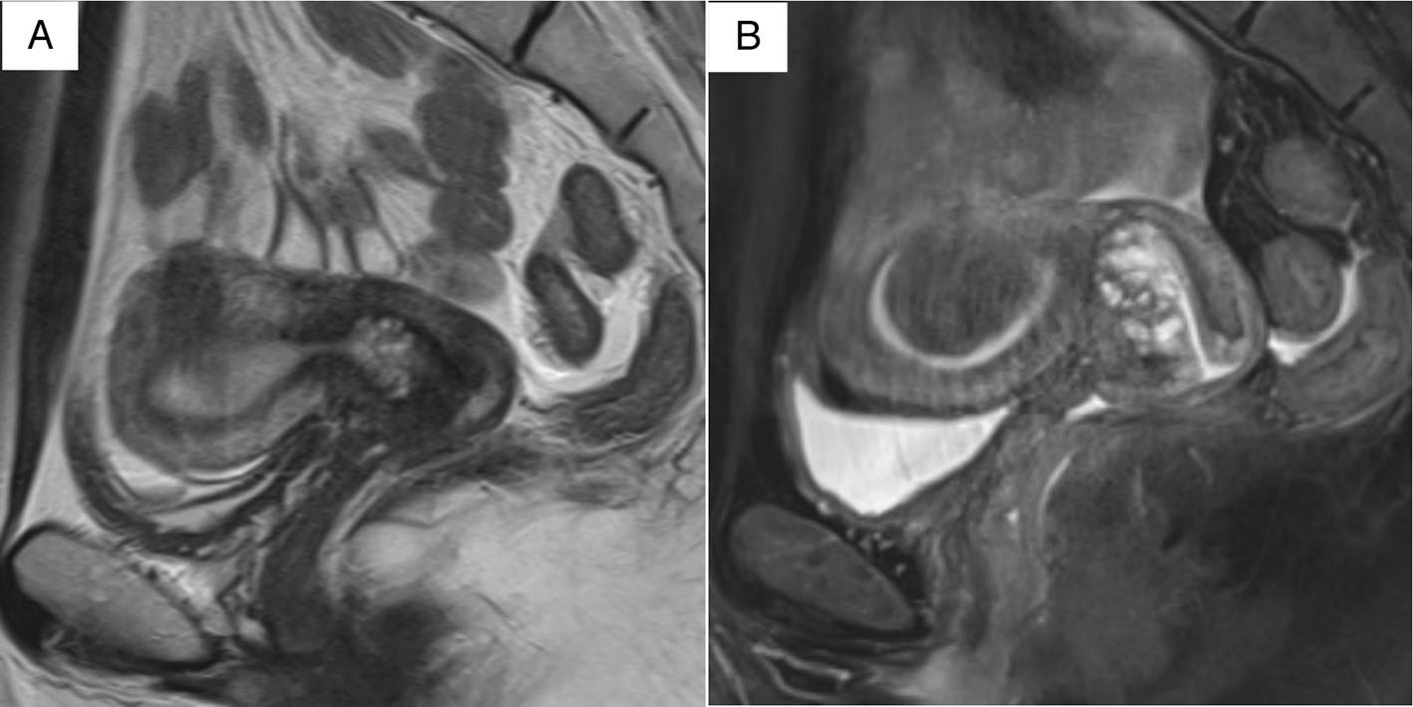
**A** Sagittal T2-weighted (T2WI) image of 34-year-old woman with lobular endocervical glandular hyperplasia shows tiny cysts in the upper part of the cervix. This lesion was classified as the “microcystic pattern.” **B** Sagittal fat-suppressed T2WI of the same patient at the age of 41 years shows that the lesion is composed of some small cysts (< 5 mm) and solid components in the central part of the lesion, surrounded by relatively large cysts (≥ 5 mm). This lesion was classified as the “typical cosmos pattern.” The lesion also increased in size

**Fig. 6:**
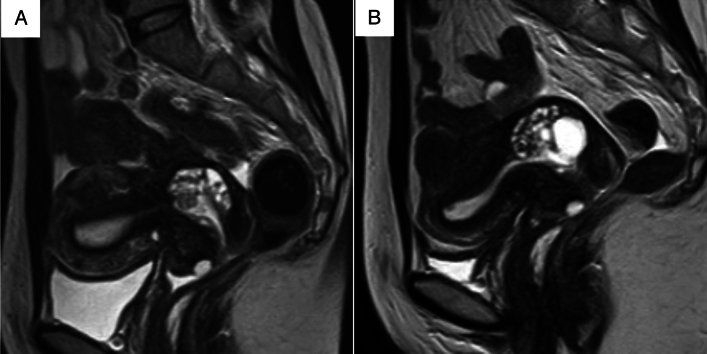
**A** Sagittal T2-weighted (T2WI) image of a 45-year-old woman with lobular endocervical glandular hyperplasia showing a cystic aggregate lesion in the upper part of the cervix. The lesion is composed of some small cysts and solid components in the central part of the lesion, surrounded by relatively large cysts. This lesion was classified as the “typical cosmos pattern.” **B** Sagittal T2WI of the same patient at the age of 51 years (premenopausal) shows a cystic aggregate lesion in the upper part of the cervix. The lesion has increased in size, but the typical cosmos pattern remained unchanged

**Fig. 7:**
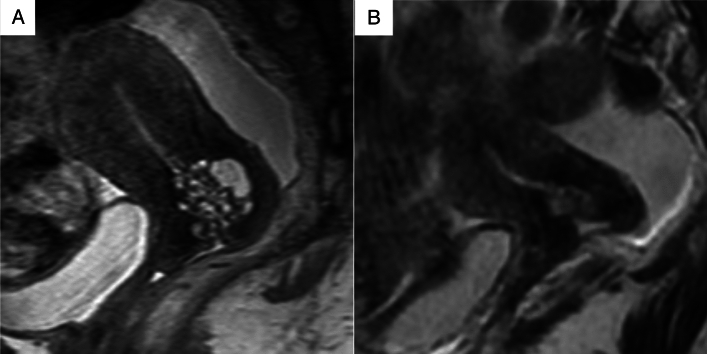
**A** Sagittal T2-weighted (T2WI) image of a 55-year-old woman (premenopausal) with atypical lobular endocervical glandular hyperplasia showing a cystic aggregate lesion in the upper part of the cervix, with some small cysts and solid components in the central part of the lesion, surrounded by relatively large cysts. This lesion was classified as the “typical cosmos pattern.” **B** Follow-up of this patient at the age of 63 years (postmenopausal) shows remarkable shrinking of the lesion and the typical cosmos pattern changed to a microcystic pattern

**Fig. 8:**
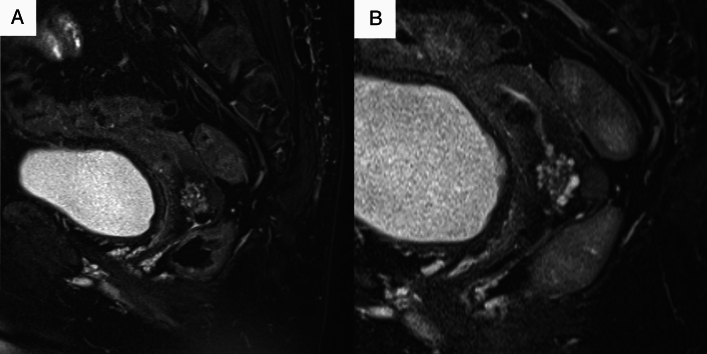
**A** Sagittal T2-weighted (T2WI) image of a 64-year-old woman (postmenopausal) with atypical lobular endocervical glandular hyperplasia showing a cystic aggregate lesion in the upper part of the cervix with numerous small cysts. This lesion was classified as the “microcystic pattern.” **B** Follow-up of this patient at the age of 66 years (postmenopausal) shows that the lesion did not change in size, and the microcystic pattern persisted

Changes in the morphologic MRI findings of LEGH during long-term follow-up are summarized in Fig. 4. Among 12 patients who underwent the initial MRI in their 30 s or at the age of 40 years, five patients showed the typical cosmos pattern in the initial MRI. Of the remaining seven patients, a non-cosmos pattern in two patients changed into the typical cosmos pattern in the MRI just before surgery (Fig. 5), whereas the non-cosmos pattern persisted in the remaining five patients. Among the 13 patients who underwent the initial MRI from the age of 41 years to pre-menopause, 10 of 13 (76.9%) patients showed the typical cosmos pattern in initial MRI, which remained unchanged in the follow-up MRI (Fig. 6). Four patients underwent follow-up MRI across menopause. In all four patients, the premenopausal MRI findings demonstrated the typical cosmos pattern. In one patient with aLEGH, the MRI findings changed from the typical cosmos pattern to a microcystic pattern after menopause (Fig. 7). The MRI findings in one case of aLEGH that was followed up after menopause remained unchanged (Fig. 8).

## Discussion

Our results revealed that the volume and MRI findings of LEGH vary significantly depending on the age group. In a study of non-operative cases, Kobara et al. [10] suggested that suspected LEGH lesions shrink in about half of the cases after menopause. Our study on patients with surgically-removed and pathologically-proven LEGH found that the median volume of the LEGH lesions after menopause was significantly smaller than that immediately before menopause. The above findings suggests that LEGH may be strongly influenced by hormones, particularly estrogen. The epithelium of LEGH is negative for estrogen receptors, but spindle-shaped cells in the stroma are estrogen positive [12], suggesting that serum estrogen levels are likely to be involved in LEGH shrinkage. The changes in the size of LEGH may be caused by an increase or decrease in the number of epithelial and stromal cells in the lesion. They may also be influenced by the mucin secretion capacity and amount of mucus stored in the cysts. Notably, our results revealed that the median volume of LEGH lesions in patients in their 30 s was smaller than that in patients just before menopause. Therefore, LEGH is characterized by a gradual increase in the lesion size from the 30 s until just before menopause, although the reason is unclear. Perhaps the LEGH size is dependent on the mucus-producing and mucus-retaining capacity of the lesions. The cysts that make up the LEGH lesion store mucus, and their size depends on their mucus-producing capacity. For reasons that remain unclear, the mucus-producing capacity of LEGH tends to increase until just before menopause. The postmenopausal shrinkage may be due to a hormone-dependent decrease in the mucus production capacity. Because of this feature, it seems impossible to distinguish between LEGH and aLEGH in terms of the LEGH volume.

As previously reported [7, 8], the typical cosmos pattern is the most representative MRI finding of LEGH, and it accounted for the largest proportion in each age group. However, the proportion varied by age group. In group A (31–40 years), the typical cosmos pattern was seen in 60% of the patients, but it was detected in only 30% of the initial MRIs of patients who underwent follow-up. Two of seven patients demonstrated a change from the non-cosmos pattern to the typical cosmos pattern during follow-up. Therefore, it can be concluded that the MRI pattern detected in group A (31–40 years) may potentially change to the typical cosmos pattern even if it was not initially the cosmos pattern. The first MRI finding of LEGH may not be the typical cosmos pattern. In addition, since the percentage of the typical cosmos pattern was very high in patients belonging to group B (41–50 years) and group C (> 50 years, pre-menopause), the cosmos pattern may be the mature form of LEGH. Importantly, the proportion of the typical cosmos pattern clearly decreases postmenopause compared to pre-menopause. The typical cosmos pattern was observed in only 29.2% of postmenopausal patients, which was the same percentage as the microcystic pattern. Thus, in postmenopausal patients, the representative MRI findings of LEGH can be described as the typical cosmos pattern and microcystic pattern. Omori et al. [9] proposed the raspberry type as a characteristic MRI finding of LEGH in postmenopausal cases, and our study also confirmed an increased proportion of raspberry-type-like microcystic pattern in postmenopausal patients. In particular, atypical LEGH, which exhibited the typical cosmos pattern before menopause, changed to a raspberry-type-like microcystic pattern after menopause. Based on these findings, it is likely that some lesions with microcystic pattern after menopause are the result of altered cosmos pattern. Omori et al. reported that the raspberry-type pattern was more common in atypical LEGH [9]. However, the trends in the changes in MRI findings with age were similar in the LEGH and aLEGH groups, and no significant differences were detected. Hence, changes in the MRI findings during follow-up are not useful for differentiating LEGH from aLEGH. However, in one case where the MRI findings changed from the typical cosmos pattern to a small cystic pattern, it was unclear at what point the lesion progressed to atypical LEGH.

Furthermore, the transition from the typical cosmos pattern to a microcystic pattern may be related to the mucus production and retention capacity of the lesion as it ages. The relatively large cysts at the periphery of the typical cosmos pattern (corresponding to the ‘petals’ of the cosmos flower) are thought to represent areas with substantial mucus retention. A shift to a microcystic pattern implies a reduction in the size of these peripheral cysts, which in turn suggests a decrease in the amount of mucus retained within the lesion. This issue requires further pathologic and radiologic contrast analyses, while taking age into account.

In this study, we did not find any differences between the LEGH and aLEGH groups in terms of the volume and MRI findings, but differences were detected between the two pathologic groups in two respects. First, the two groups differed in the obstetric history. The proportion of patients who had not experienced a pregnancy was significantly higher in the LEGH group. It is possible that some factor related to pregnancy is involved in the development of atypical LEGH. However, the possibility of selection bias cannot be ruled out due to the small number of cases. Slight differences in age distribution between the two groups may also have affected the difference in findings. Further studies are needed to clarify this difference. Secondly, there was a slight difference in the concordance rate of MRI findings between the two radiologists for the LEGH and aLEGH groups. The LEGH group had a higher agreement rate. It is possible that the MRI pattern was difficult to identify in more cases in the aLEGH group compared to the LEGH group. Accordingly, cases in which the MRI findings are difficult to evaluate may be aLEGH. However, this issue needs further investigation.

There were some limitations of our study. First, the MRI parameters varied in the study. Notably, the slice thickness and other image parameters may affect the MRI findings. However, this limitation was unavoidable because the data were obtained over a long period in this multicenter retrospective study. Second, the intervals between the follow-up MRIs were not constant. It is possible that MRI performed at close regular intervals may have captured the time point when the LEGH changed to aLEGH, which may have helped to differentiate LEGH from aLEGH in terms of the size and MRI findings. However, this limitation was also unavoidable because the data were obtained over a long period in this multicenter retrospective study.

In conclusion, the imaging findings of LEGH change with age. LEGH shows the highest median volume just before menopause and the lowest median volume after menopause. The MRI pattern may also change with age; from the 30 s to premenopausal years, the proportion of the typical cosmos pattern increases with maturation, whereas postmenopause, the proportion of the typical cosmos patterns decreases and the microcystic pattern emerges as a characteristic postmenopausal finding of LEGH. Therefore, during the 30 s and premenopausal period, LEGH can be recognized based on the typical cosmos pattern; however, caution is needed after menopause as LEGH cases with patterns other than the typical cosmos pattern become more common. Unfortunately, we found that distinguishing between LEGH and aLEGH based on serial MRI findings is challenging. In addition, the expansion or shrinkage of lesions is not associated with whether the condition is LEGH or aLEGH.
